# Mucinous Carcinomatosis: A Rare Association between an Ovarian Tumor and an E-GIST

**DOI:** 10.1155/2018/6897372

**Published:** 2018-01-11

**Authors:** Hugo Palma Rios, André Goulart, Pedro Leão

**Affiliations:** ^1^General Surgery Department, Hospital de Braga, Braga, Portugal; ^2^Surgical Sciences Research Domain, Life and Health Sciences Research Institute (ICVS), School of Health Sciences, ICVS/3B's-PT Government Associate Laboratory, University of Minho, Braga, Portugal

## Abstract

Pseudomyxoma peritonei (PMP) and extragastrointestinal stromal tumors (E-GISTs) are both rare entities. Most of the time, PMP is associated with an appendiceal tumor. An ovarian mucinous tumor can mimic appendiceal metastases. E-GIST is a mesenchymal tumor that can arise from the omentum, retroperitoneum, mesentery, or pleura. We present a case of an 87-year-old woman with mucinous carcinomatosis and acute intestinal occlusion submitted to an emergency laparotomy. She has found to have a borderline mucinous tumor of the ovary from the intestinal type with several lesions of pseudomyxoma peritonei and an E-GIST from the epiploons retrocavity (intermediated risk). In the literature, no relation was found between these two rare tumors. E-GIST was an incidental finding in the context of a mucinous carcinomatosis.

## 1. Introduction

Mucinous carcinomatosis or pseudomyxoma peritonei (PMP) is an accumulation of mucinous fluid in the abdominal cavity. Most cases are caused by mucin-producing tumors of the appendix and ovary [1]. Mucinous tumors represent 32% of all epithelial borderline ovarian tumors, the intestinal type being the most common (90%) [2]. Gastrointestinal stromal tumors (GISTs) account for only 2% of all gastrointestinal neoplasms and are the most common mesenchymal tumor of gastrointestinal type [3]. More than 95% of GISTs express the KIT protein (CD117). Extragastrointestinal stromal tumor (E-GIST) develops outside the gastrointestinal (GI) tract, such as the omentum, mesentery, and retroperitoneum [4]. The incidence of E-GIST is estimated to be 10% of all GISTs, but the pathologic characteristics and clinical outcomes are still on debate due to its rarity [5].

## 2. Case Report

We present the case of an 87-year-old woman with a relatively good performance status brought to the emergency room with progressive asthenia, weight loss, and abdominal distension for the last two months. She had a previous diagnosis of diabetes mellitus under oral antidiabetics and hypercholesterolemia and no previous surgical procedures. On physical examination, she was pale, emaciated, afebrile, and with abdominal distension for ascites without tension. Edema in the lower members was also present.

On laboratory workup, a minor microcytic anaemia was detected; renal function, liver markers of cytolysis, and inflammatory markers were normal. Tumor markers, such as carcinoembryonic antigen and carbohydrate antigen 19.9 and 125, were within normal range. She was admitted for further workup.

**Figure 1:**
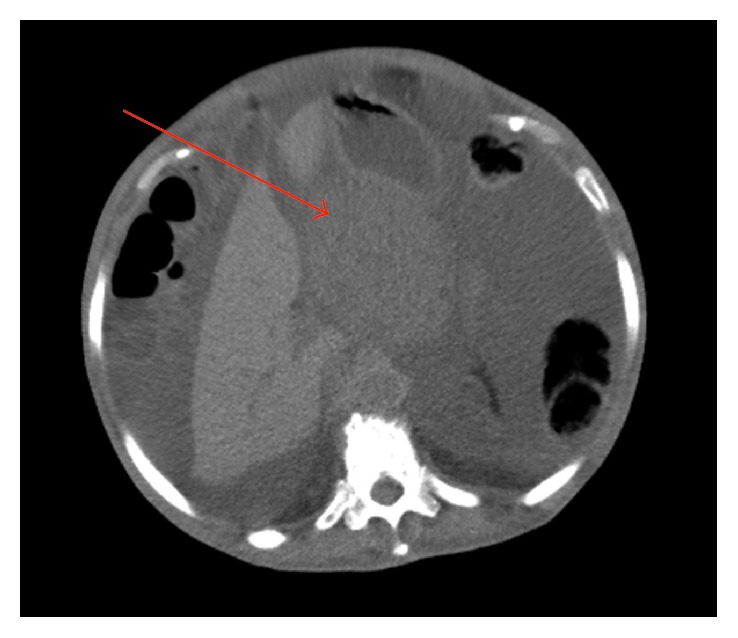
CT scan showing a lobulated mass in front of the pancreas measuring 8.5 × 9.5 cm (arrow), signs of chronic liver disease, and high-volume ascites.

Abdominal ultrasound described a mass between the stomach and pancreas with about 9 cm and large volume ascites. Abdominal CT scan described a lobulated mass anterior to the pancreas with 8.5 × 9.5 cm, signs of chronic liver disease, and confirmed large volume ascites (Figure 1). Endoscopic study of both high and low GI tract was normal. A paracentesis revealed a mucinous content, negative in microbiologic cultures. Cytology and biochemical analysis were inconclusive.

**Figure 2:**
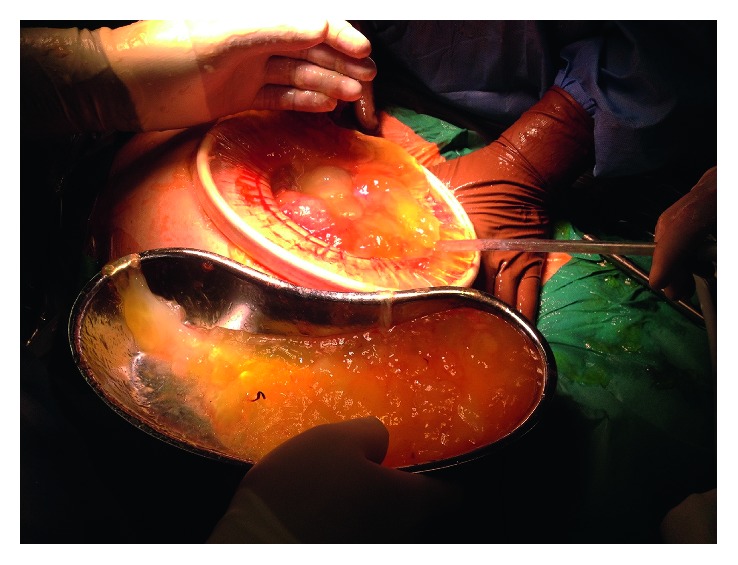
High volume of mucinous ascites.

**Figure 3:**
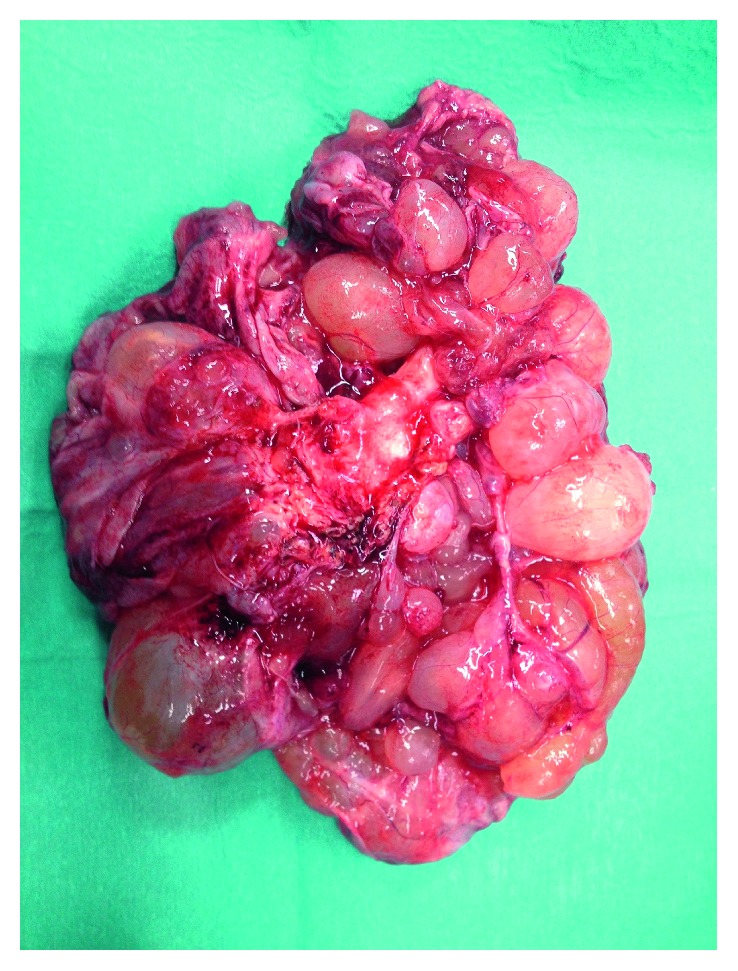
A multiloculated mass in the left ovary.

**Figure 4:**
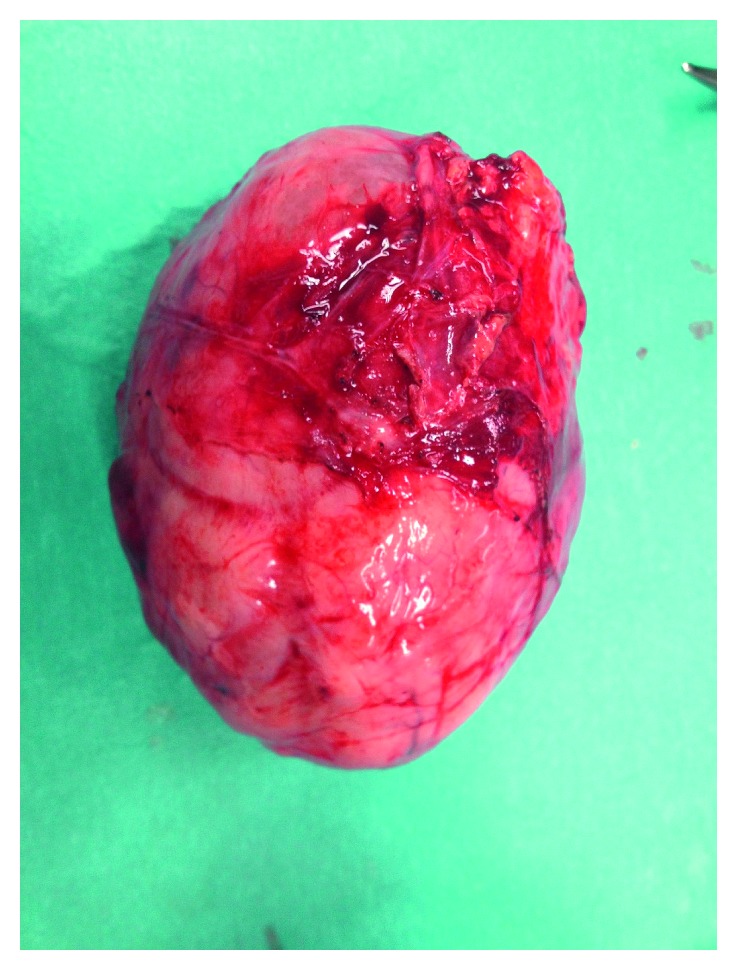
A lobulated mass in the epiploons retrocavity near the pancreas.

**Figure 5:**
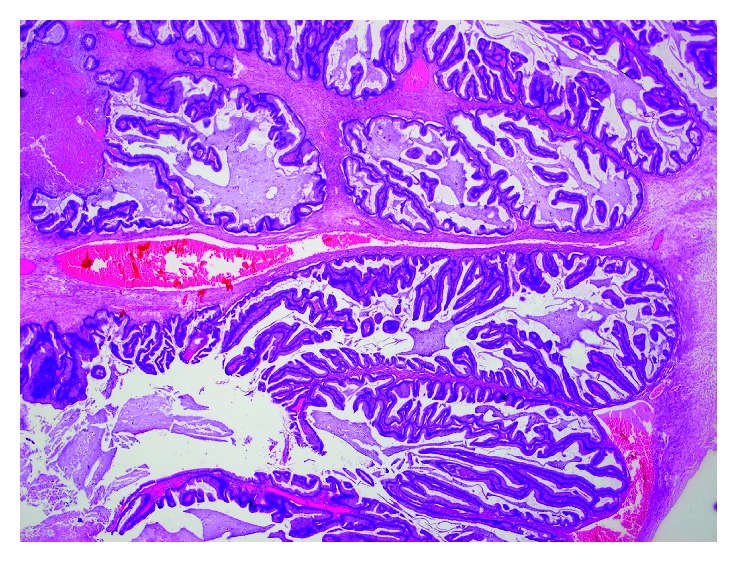
Borderline mucinous tumor of the ovary (intestinal type; HE 20x).

**Figure 6:**
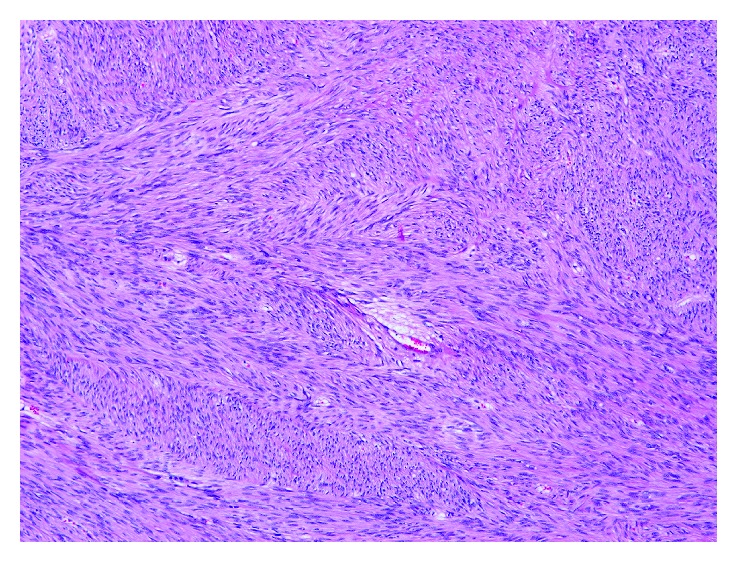
Spindle cell lesion with eosinophilic cytoplasm and elongated nucleus (E-GIST from the epiploons retrocavity; HE 100x).

One week after admission, the patient had persistent vomiting and dyspnea caused by increased abdominal distension. An emergency laparotomy was performed. A total of 12 L of mucinous ascites (Figure 2) was removed, and two lesions were identified. The first lesion was a multiloculated mass in the left ovary (Figure 3), and the second was a lobulated mass in the epiploons retrocavity near the pancreas (Figure 4). Both tumors were totally removed. No macroscopic changes were found in the appendix. Pathology revealed brownish membranous areas and multiple cystic formations with gelatinous and yellowish content suggestive of borderline mucinous tumor of the ovary of the intestinal type with broken capsule and several lesions of pseudomyxoma peritonei (pT1c Nx (FIGO IC)) (Figure 5) [6, 7]. An appendiceal origin or a teratomatous component was excluded. The second tumor was an E-GIST from the epiploons retrocavity, with a low mitotic index (2/50 HPF). Immunohistochemically, the tumor cells were positive for CD34, DOG1, and CD117 but negative for S100 protein (Figure 6). The patient had full recovery from surgery without any complications. After a multidisciplinary discussion, no adjuvant therapy was performed, and the patient was discharged to a palliate care facility. Within the two years of follow-up, the patient did not present any evidence of recurrence.

## 3. Discussion

Pseudomyxoma peritonei is a clinical condition more frequently originated in a perforated mucinous tumor of the appendix. The accumulation of gross mucinous ascites can lead to adhesions and cause intestinal obstruction.

The clinical course is insidious, and multiple recurrences are common. Surgery with cytoreductive therapy and intraperitoneal chemotherapy is the approach with better outcomes described.

A primary ovarian tumor rarely causes PMP and the most frequently associated subtype is the mature teratoma [8, 9]. It is important to differentiate a primary tumor from those arising from the appendix and a mucinous tumor from the ovary since they can share some histological aspects [10, 11].

Some available data suggested that “borderline” mucinous tumor from the ovary can be originated from a primary appendiceal lesion. When the lesion presents benign and borderline areas and smooth surface and is unilateral and limited to the ovary, these characteristics point to ovarian origin. Pathologists use a panel of immunohistochemical markers for ovarian origin (CK7, CK20, and CDX-2) even though some combinations can be shared with other tumors (pancreatobiliary, upper gastrointestinal tract, lung, and breast). Metastatic lesions are more common and usually present as small diffuse lesions with an infiltrative pattern of stromal invasion [12].

In the case presented above, the appendix was not resected because it did not show abnormal appearance and a unilateral lesion in the ovary was resected.

The other tumor removed was characterized as an E-GIST, a mesenchymal tumor that originates from interstitial cells of Cajal and arises outside the GI tract. While some authors consider that E-GISTs may originate as primary tumors from mesenchymal stem cells of the greater omentum and mesentery, others consider that these tumors derive from the gastrointestinal tract like the other GISTs and may represent a metastatic stage [13].

The tumor could be asymptomatic for a months or years and sometimes can be palpable. The presence of symptoms is associated with a large lesion. Most of cases are diagnosed through CT scan. The lesions are usually single, well delimited, and with low density [3].

Yi et al. [14] published a revision of 51 patients with E-GIST in South Korea. The most common location was the mesentery (*n* = 15) followed by the retroperitoneum (*n* = 13) and omentum (*n* = 8).

Several case series studied grading systems for these tumors. Usually, tumor size and mitotic rate are used to classify the other GISTs. As we discussed above, E-GISTs are more frequently symptomatic only when they are already large masses. Therefore, size is not probably as useful for grading as for the other GISTs. Mitotic rate, cellularity, and Ki67 expression seem to be the most significant prognostic factors. In most cases, E-GISTs are large tumors associated with high proliferative rates, sometimes with lymph node and distant metastasis, which entails a worse prognosis [15, 16].

Yamamoto et al. [5] defined three categories: the high-risk group (≥5/50 HPF with ≥10% Ki67), the intermediate-risk group (≥5/50HPF with <10% Ki67 or <5/50 HPF with ≥10% Ki67), and the low-risk group (<5/50 HPF with <10% Ki67). This classification can be useful to determine the indication for adjuvant therapy.

Surgical resection is the treatment of choice. After the surgery, patients are selected for treatment with inhibitors of tyrosine kinase that follows the NIH criteria: tumor size, mitotic rate, and anatomic location. Our patient's tumor was classified as moderate risk, and no adjuvant treatment was given.

No relationship between a borderline ovarian mucinous tumor and an E-GIST was found in the literature [17, 18]. In the same way, pseudomyxoma peritonei was not related with any type of GIST. From our point of view, these two tumors presented as synchronous and unrelated. Looking for the characteristics of the E-GIST, this tumor probably developed for a long time before and was an incidental finding in the context of a mucinous ascites from another primary tumor.
